# Technologically assisted intensive home treatment: feasibility study

**DOI:** 10.3389/fpsyt.2023.1196748

**Published:** 2023-07-27

**Authors:** Asaf Caspi, Dana Tzur Bitan, Ofir Halaly, Ofri Hallaly, Avraham Friedlander, Galia Barkai, Eyal Zimlichman, Orit Stein, Mordechai Shani, Ziv Amitai, Tsofiya Ansbacher, Mark Weiser

**Affiliations:** ^1^The Drora and Pinchas Zachai Division of Psychiatry, Sheba Medical Center, Ramat Gan, Israel; ^2^Department of Behavioral Sciences, Ariel University, Ariel, Israel; ^3^Shalvata Mental Health Center, Hod Hasharon, Israel; ^4^Central Management, Sheba Medical Center, Tel-Hashomer, Ramat Gan, Israel; ^5^Sackler Faculty of Health, Tel-Aviv University, Tel-Aviv, Israel; ^6^Maccabi Health Services, Tel Aviv, Israel

**Keywords:** psychiatric hospitalization, admission, technology, technologically assisted intensive home treatment, psychiatric services

## Abstract

**Introduction:**

In recent year, many attempts have been made to provide patients with alternatives to psychiatric hospitalization during acute distress. Although several hospitalization alternatives have been offered, most of them still require patients to be distanced from their families, friends, and the social environment.

**Methods:**

In this report we describe the implementation of a novel approach to psychiatric care termed “Technologically assisted Intensive Home Treatment”, where patients arriving to emergency settings are directed to home care with technological aids that enable close monitoring and ongoing contact with their therapists.

**Results:**

We describe the rationale and treatment principles of the treatment, and provide an elaborative description of the implementation process during the first year of implementation.

**Discussion:**

Additional attention is given to factors associated with early dropout from the program, in order to inform readers of predictors to optimal care. Limitations and directions for future research and practice are discussed.

**Clinical Trial Registration:** The study was registered in the database of clinical trials (registration number SHEBA-19-6555-MW-CTIL) and in the Ministry of Health (registration number MOH_2022-08-22_011992).

## Introduction

Psychiatric inpatients settings are considered the most routine and traditional route to provide care to patients with acute mental outbreaks. Nonetheless, in recent years attention has been brought to the potential adverse effects of admission to psychiatric hospitals for individuals with severe mental disorders. These adverse effects are reported to include self-esteem impairment (1), social maladaptation (2) and symptomatic deterioration (3). Studies which explored reasons for patients’ non-adherence with the psychiatric system have reported lack of self-control, adverse experiences with the treating staff (4, 5), feeling foreign from the therapeutic system (6) and social and self-stigma (7, 8) as some of the potential reasons for reduced compliance. These effects, as well as economical considerations pertaining to inpatient costs, have led scholars, clinicians and policy makers to explore alternatives to the traditional model of psychiatric hospitalization so as to mitigate these adverse consequences.

Alternative forms of psychiatric care during acute states have been established relatively early. In the late 1960s and early 1970s the Soteria model was suggested by Lauren Mosher in California (9), as means to address the needs of young people with severe mental disorders and specifically facilitate community integration and adjustment to the neighborhood’s social norms. Crisis homes, which include community facilities staffed 24/7 by clinical staff and provide a short-term stay for individuals with mental disorders has also been described (10, 11). Nonetheless, some of the models were struggling to balance medical and social models of care, or were more fitted to specific populations (9, 10, 12). More importantly, most of them require patients to undergo their crisis away from their close and familiar environment.

The use of technology during the COVID-19 pandemic has provided further evidence for the potential of technological aids to act as therapeutic means for patients, even in their own homes. Intensive Home Treatment (IHT) typically involves treatment in patients’ home and a facilitation of a decision-making process. During home treatment, a professional team visits the patient more than once a day (13). In a recent study comparing the effectiveness of home-based care to inpatient hospitalization, similar effectiveness in improving clinical symptoms was observed, along with a reduction in hospitalization days over the 2 years following the intensive home treatment (14). Studies aimed to explore the effectiveness of therapies provided online have demonstrated their non-inferiority to face-to-face treatments, thus supporting the notion that treatments given during hospitalization may be provided online (15). Studies that focused on treatment processes have indicated that the quality of the therapeutic alliance created in online therapy is similar to the quality of face-to-face therapy (16, 17). In medical settings, online medical counseling was found to be as effective as face-to-face treatment in terms of quality of communication and accessibility to treatment (18). Furthermore, Hickson et al. (18) suggested that online care may lead to increased access to care by reducing patients’ travel costs. These findings suggest that technology might assist clinicians and patients to overcome the barriers and challenges of psychiatric hospitalization, by providing home care through the utilization of online services.

In 2019 clinicians in Sheba Medical Center in Israel initiated the implementation of “Technologically assisted Intensive Home Treatment”. The project was initiated by the support of the Maccabi and healthcare services. The idea of remote hospitalization is that individuals with severe mental outbreaks will be treated at home using technological aids. The service was aimed to ameliorate stigma and significantly improve patients and families’ experiences with the public mental health system. To reach this aim, a complex technological system managing multi-disciplinary therapeutic interventions was developed so as to allow access to treatment records by clinicians, as well as to monitor patients’ physical status *via* sensors and self-report measures. Because the model does not depend on location, the remote service allowed the treating team to be continuously available to accompany the patient. The presence of a primary care giver, a family relative or close friend, who is with the patient most of the day, helped the treating staff to monitor potential risk. During the treatment, information was collected mainly through a smart watch, in order to assist in clinical assessments and decision making. The services followed the principles of continuity of care, therefore, the same professional staff accompanied the patients throughout the treatment at varying levels of intensity, depending on the patient’s needs.

In this article we present the implementation process of the first technologically assisted intensive home treatment in Israel. Studies suggest that the effectiveness of novel treatments depends largely on the implementation process, with effective implementation associated with better therapeutic outcomes (19). Thus, this study is aimed to explore the benefits and drawbacks of technologically assisted intensive home treatment, and provide a full account of the challenges faced by the treating staff. Specifically, the following objectives were pursued: (a) providing a full description of the treatment model; (b) describing the nature of the patients participating in the implementation process; (c) discuss terms and conditions needed to reach therapeutic success in this model; and (d) provide directions for future research and suggestions to other groups which may be interested in implementing such services.

## Methods

### Basic principles of technologically assisted intensive home treatment

The basic model of the technologically assisted intensive home treatment is based on a sequence of interventions which overall can last for up to 2 years. This sequence starts with assessment of suitability, followed by remote hospitalization. During remote hospitalization patients receive therapeutic interventions, assessments and online monitoring, as well as in-person online meetings held in fixed intervals. The treatments are personal, conducted by a multi-professional team, and are customized to fit the needs of the patient and his primary caregiver; All treatments are managed by a case manager, and include psychiatric medical follow-up, psychological therapy and social assistance by a social worker, as well as continuous monitoring of behavioral and physical health. Continuous measurements of behavioral data such sleep patterns, level of physical activity, medication adherence, and vital signs including physiological measures (body temperature, blood pressure, and heart rate) was performed, and patients had mobile phone reminders to take medications and attend follow-up appointments. Therapists could also utilize the system for individual and group interventions through video conferencing. The service technology was based on a tablet with a dedicated application developed by Datos, a smartwatch measuring blood pressure and heart rate, a thermometer, a saturation monitor. The Datos application also enabled messaging communication and video calls with the healthcare team. The smartwatch also monitored sleep patterns and physical activity through step tracking. Blood pressure, temperature, and oxygen saturation data were transmitted to the Datos application on the tablet via Bluetooth.

Staff was compiled of a multi-disciplinary professional team that includes a psychiatrist, psychologist, social worker, nurse, occupational therapist and nutritionist. Patients participating in the program had 24/7 telephone availability of a psychiatric nurse. Data security measures are utilized to safeguard patient’s confidentiality and privacy. During hospitalization, the degree of suitability of the patient to the service is constantly examined, so as to avoid potential risk. The medical doctor prescribes medications in the patient’s medical record at Sheba Medical Center, which automatically transfers to a dedicated application accessible to the patient. Afterwards, family physician in the community submits a request to the health insurance fund for dispensing the medications. In cases of low compliance with medications, a smart medication dispenser sends reminders for intake and alerts the healthcare team.

### Treatment procedure

The acute stage of treatment includes high intensity care, and usually lasts 4–6 weeks. The first stage is initial assessment, where patients are referred to remote hospitalization after an examination in a psychiatric emergency room, a psychiatric clinic (community or hospital) or during a psychiatric hospitalization that does not exceed 2 days. After starting the program, the comprehensive care package is offered and is supported, if necessary, by community caretakers. Patients are equipped with a home kit with full communication platform to make video calls, send two-way messages, collect information through sensors, reminded to take medication and schedule follow-up appointments. The kit is based on a tablet computer. Significant clinical exacerbations, transfer to full hospitalization, incidents of self-harm and suicide and events of new physical illness are documented and reported to the risk management department at Sheba Medical Center.

### Criteria for inclusion and exclusion

The service is designated for patients who need urgent psychiatric hospitalization, as clinically evaluated by a senior psychiatrist. Patients starting full hospitalization are eligible to transfer into the home care. Patients with one of the following diagnoses can join the services: psychotic disorder in its early stages (up to 5 years from the first psychotic episode), mood disorders, postpartum depression, or psychosis. The inclusion of a patient in the service is conditioned on the immediate availability of a primary caregiver such as a parent or spouse with good support capacity. Patients with one of the following definitions will not be included in the service: patients with immediate risk as a result of the mental illness to aggression or suicide, patients admitted for forced hospitalization, current drug or alcohol abuse, low levels of response to treatment, developmental intellectual disability or other disability which might cause difficulty in technological operation, significant physical illness requiring hospitalization, refusal to take part in treatment as part of the service.

## Results

Table 1 presents the demographic characteristics of the patients admitted to the service during the first year of implementation. The total sample included 58 people, 69% were women. The average age was 37.52 (SD = 15.99). The most common diagnostic category was mood disorders with a frequency of 43.9% (*n* = 25), personality disorders with a frequency of 26.3% (*n* = 15), anxiety disorders with a frequency of 15.8% (*n* = 9) and schizophrenia and psychosis with a frequency of 14% (*n* = 8).

**Table 1:** Demographic and clinical characteristics of the study sample.

Characteristics	Technologically assisted intensive home treatment (*N* = 57)
Age (M, SD)	37.52 (15.99)
Gender (% females)	40 (69)
Diagnoses	
Personality disorder (%)	15 (26.3)
Anxiety disorder (%)	9 (15.8)
Mood disorder (%)	25 (43.9)
Schizophrenia and psychosis (%)	8 (14)

Table 2 presents the clinical outcomes of the technologically assisted intensive home treatment. The average number of days in technologically assisted intensive home treatment was 193.17 (SD = 208.6). Of all participants, 59% (*n* = 34) completed hospitalization and 41% (*n* = 24) dropped out prior to official discharge. Of those who dropout from hospitalization, 33% (*n* = 8) dropped out during the first month. The most common reason for leaving in the first month is lack of cooperation (*n* = 5, 62%, including transfer to another treatment), aggravation or lack of improvement (*n* = 2, 25%), and dropout due to technical reason (*n* = 1, 12%). The most common reason for leaving after the first month is aggravation or lack of improvement (*n* = 9, 56%), dropout due to lack of cooperation (including transfer to other treatment *n* = 3, 19%) and dropout due to technical reason (*n* = 4, 25%). Of the total sample of dropouts, the majority (62%) moved to full hospitalization, with 80% of them transferring to involuntary hospitalization and 20% moving to consensual hospitalization. These numbers demonstrate the main complexity of technologically assisted intensive home treatment in balancing acute states.

**Table 2:** Outcomes of the first year of implementation.

Category	Sub-category	*N* (%)
Overall time in service (M, SD)		193.17 (208.6)
Status	Completes (%)	34 (58.6)
	Dropped (%)	24 (41.4)
Dropped time	In the first month (%)	8 (33.3)
	After the first month (%)	16 (66.6)
Reasons for leaving service in the first month (*n* = 8)	Aggravation or lack of improvement (%)	2 (25.0)
	Lack of cooperation (%)	5 (62.5)
	Release on a technical or other (%)	1 (12.5)
Reasons for leaving service after the first month (*n* = 16)	Aggravation or lack of improvement (%)	9(56.2)
	Lack of cooperation (%)	3 (18.7)
	Release on a technical or other (%)	4 (25.0)
Overall number of patients to full hospitalization (*n* = 15)	Involuntary (%)	12 (80)
	Consensual (%)	3 (20)

Table 3 presents the demographic and clinical characteristics of patients who dropped out of the service (*n* = 24). The average age of the dropouts is 35.71 (SD = 18.20). Out of all the dropouts, 17 are women (70.8%). Average days in service is 128.83 (SD = 113.31). The most common diagnosis among the dropouts is Anxiety/Mood disorder with about 50% (*n* = 12), followed by Personality disorder with 29.9% (*n* = 7), and the disorder with the lowest frequency among the dropouts is Schizophrenia with 20.8% (*n* = 5). The most frequent staff position for the dropouts is that the patient needed full hospitalization (50%, *n* = 12), followed by release due to various reasons with 25% (*n* = 6), that the patient was unfitted to service with 12.5% (*n* = 3) or should continue treatment (12.5%, *n* = 3).

**Table 3:** Characteristics of patients who dropped out of the service (*n* = 24).

Characteristics		
Age	M, SD	35.71 (18.20)
Sex	% Female	17 (70.8%)
Diagnosis	Personality disorder	7 (29.2%)
	Mood/anxiety disorder	12 (50.0%)
	Schizophrenia	5 (20.8%)
Total days in service	M, SD	128.83 (113.31)
Team position	Unfitted to service	3 (12.5%)
	Needs full hospitalization	12 (50%)
	Should continue treatment	3 (12.5)
	Release due to various reasons	6 (25.0%)

## Discussion

This study was aimed to provide a full account of the first implementation of technologically assisted intensive home treatment in Israel, based on the first 58 patients who entered this program at Sheba Medical Center in Israel. To the best of our knowledge, this is the first program in Israel and worldwide to implement full psychiatric hospitalization while monitoring the psychological, medical, pharmacological, occupational and social treatment of patients using technological aids.

The results of the first year of recruitment to the program indicated that the vast majority of patients completed the treatment program. Nonetheless, 24 patients, which constitute 41% of the total sample, did not complete the treatment. Eight patients also failed to complete the first month of hospitalization. Several hypotheses can be made to explain these attrition rates. An analysis of the characteristics of the patients who dropped out of the program indicates that staff position was that these patients needed full hospitalization. Furthermore, most of the patients leaving the program did so due to aggravation in their clinical state, or due to lack of cooperation with the offered treatment program. The implementation process might have also differentially affected patients with different clinical characteristics. For example, it is possible that patients with severe anxiety and additional underlying pathology could not tolerate the experiential nature of the service and therefore were more likely to dropout. Thus, it is possible that for some patients, home environment is not sufficient for acute stabilization. Studies conducted in full psychiatric hospitalization settings demonstrate the importance of having clinical staff with the patient 24 h a day. (20) explained that the presence of the care staff 24 h a day makes it possible to anticipate future crisis events and prevent them. It is therefore possible that for some patients, the 24-h presence of professionals is paramount.

An analysis of the main characteristics of the dropouts from the technologically assisted intensive home treatment reveals that most of the dropouts were women with mood or anxiety disorder, followed by personality disorder. Schizophrenia had the lowest frequency of dropout, although the number of patients with schizophrenia recruited to the service was low to begin with. The high frequency of dropouts with mood and anxiety disorders may be associated with an underlying personality disorder (21), which may have been difficult to handle without a designated treatment approach. Furthermore, the majority of patients who dropped out entered full hospitalization. This may be associated with the novelty of the treatment approach, as compared to the familiarity of the inpatient care. Studies indicate that the credibility of the treatment approach, the fact that it well known, affects patients’ trust and comfortless with the approach. Furthermore, Frovenholt et al. (22) found that the perceptions of credibility of the therapeutic procedure may affect both the therapeutic alliance and the treatment outcomes. It is therefore possible that once this alternative care will be disseminated, patients will be more comfortable with the treatment approach. Such an hypothesis remained to be examined in future initiatives.

The technology used in the implementation of the Technologically assisted Intensive Home Treatment included a tablet with a dedicated application developed by Datos, a smartwatch measuring blood pressure and heart rate, a thermometer, a saturation monitor. The technology was most helpful in videoconferencing and management of sleep disturbances. On the other hand, alerts of timing of medications and requests to report medication adherence was sometimes uncomfortable to some of the patients. Although no emergency alert was designated in the software, patients could call a nursing staff unit 24/7. Future developments of technologically-assisted services might use these conclusions to tailor other more adaptive functions, such as emergency button or more convenient management of patients’ medications.

As this is the first implementation of remote hospitalization using technology, it is also possible that other factors related to the implementation and dissemination process affected the outcomes of the first year. Studies indicate that the effectiveness of a new treatment depends largely on the quality of its assimilation. More specifically, it has been found that effective assimilation is associated with better treatment outcomes among mental health patients (23). DuBois et al. (24) found that programs which were monitored during implementation obtained effect sizes three times larger than programs that reported no monitoring. Moreover, Tobler (25) reported that 29% of the outcomes derived from 143 drug prevention studies were drawn from interventions that were improperly implemented, and comparisons suggested that well-implemented programs achieved effect sizes 0.34 greater than poorly implemented programs. These findings stress the importance of empirically evaluating treatment programs to improve patients’ care.

The findings of the present study have several clinical and research implications. First and foremost, the results indicate that technologically assisted intensive home treatment is a feasible alternative. Furthermore, it was evident that some patients can receive treatment in their own house, and that a continuous psychiatric care can be delivered using technological aids. This ability to provide healthcare from afar is especially important in light of the adverse social effects of psychiatric hospitalization, such as social stigma (8), and potential feelings of alienation from the inpatient system (6). The fact that patients can stay in their own home and within their own community environment fosters the social concept of inclusion and tolerance, and may also impact social norms. As the length stay in the technologically assisted intensive home care is relatively long, this service may also be considered as a service that might replace hospitalization, but then continues as remote outpatient care. Empirically, the description of the process of implementation may encourage additional scientific explorations pertaining to the conditions needed to optimize this line of treatment.

Several limitations should also be noted. First, since this is the first technologically assisted intensive home treatment in Israel, the study and treatment staff were faced with many challenges which likely affected the overall implementation process. Future studies should explore the outcomes of this implementation after full dissemination of the treatment program. This study did not include in-depth interviews with patients, caregivers and their families to assess patients’ and social partners’ level of satisfaction with this novel service. Future studies should explore whether patients and their families may have suggestions to improve the quality of technologically assisted intensive home treatment. Finally, the outburst of the COVID-19 pandemic has dramatically affected the number of patients arriving to receive acute care, and therefore also affected the overall number of patients participating in the program. Additional studies are needed to further illuminate the strengths and limitations of this treatment program. Taken together, the efforts of implementation support the feasibility of technologically assisted intensive home treatment, and provide a new horizon to inform research and clinical practice.

## Data availability statement

The original contributions presented in the study are included in the article/supplementary materials, further inquiries can be directed to the corresponding author.

## Ethics statement

The studies involving human participants were reviewed and the study was approved by the institutional review board of Sheba Medical Center in Israel (reference number: 6555-19-SMC) in January 2020. The patients/participants provided their written informed consent to participate in this study.

## Author contributions

AC, DT, OiH, and OrH conceptualized the study design and methodology, did the literature search, wrote the original draft, contributed to validation, curation, analysis, interpretation of the data, reviewed, edited, and finalized the manuscript. AF and TA contributed to validation, curation, analysis, interpretation of the data, writing, reviewing, and editing. GB, EZ, OS, MS, AZ, and MW conceptualized the study design and methodology, contributed to project administration, investigation, data acquisition, curation, validation, writing, reviewing, and editing. All authors contributed to the article and approved the submitted version.

## Conflict of interest

DT received research grants from the American Psychological Foundation and from Pfizer.

The remaining authors declare that the research was conducted in the absence of any commercial or financial relationships that could be construed as a potential conflict of interest.

## Publisher’s note

All claims expressed in this article are solely those of the authors and do not necessarily represent those of their affiliated organizations, or those of the publisher, the editors and the reviewers. Any product that may be evaluated in this article, or claim that may be made by its manufacturer, is not guaranteed or endorsed by the publisher.
